# Optimization of Digital Light Processing Three-Dimensional Printing of the Removable Partial Denture Frameworks; The Role of Build Angle and Support Structure Diameter

**DOI:** 10.3390/ma15062316

**Published:** 2022-03-21

**Authors:** Mostafa Omran Hussein, Lamis Ahmed Hussein

**Affiliations:** 1 Department of Prosthodontic Sciences, College of Dentistry in Ar Rass, Qassim University, El-Qassim, Saudi Arabia; 2 Department of Removable Prosthodontics, Faculty of Dentistry, Misr International University, Cairo, Egypt; lamis.hussein@miuegypt.edu.eg

**Keywords:** prototyping, additive manufacturing, build orientation, accuracy, printing time

## Abstract

The optimal three-dimensional (3D) printing parameters of removable partial denture (RPD) frameworks should be studied to achieve the best accuracy, printing time, and least materials consumed. This study aimed to find the best build angle and support structures’ diameter of the 3D printed (RPD) framework. Sixty (RPD) frameworks (10 in each group) were manufactured by digital light processing (DLP) 3D printing technology at three build angles (110-D, 135-D, and 150-D) and two support structures diameters (thick, L, and thin, S). Six groups were named according to their printing setting as (110-DS, 135-DS, 150-DS, 110-DL, 135-DL, and 150-DL). Frameworks were 3D scanned and compared to the original cast surface using 3D metrology software (Geomagic Control X; 3D Systems, Rock Hill, SC). Both printing time and material consumption were also recorded. Data were tested for the significant difference by one-way analysis of variance (ANOVA) test at (α = 0.05). The correlations between outcome parameters were also calculated. The 110-DL group showed the least accuracy. Significantly, the printing time of the 150-D groups had the lowest time. Material consumption of group 110-DS presented the lowest significantly statistical value. Printing time had a linear correlation with both accuracy and material consumption. Within the study limitations, the 150-degree build angle and thin diameter support structures showed optimal accuracy and time-saving regardless of material consumption.

## 1. Introduction

Recently, additive manufacturing has been widely used to produce medical and dental devices, appliances, and prostheses. This technology had several types of processing. The most common processes applied in the dentistry are vat-photopolymerization, material extrusion, material jetting, and powder bed fusion [1]. Although it shows less accuracy in fabricating dental prostheses, it is more efficient in fabricating complex structures that are hardly produced by subtractive technology [2,3,4,5,6]. Accordingly, optimization of accuracy, printing time, and material used should be considered [7,8]. Several studies were conducted to check the best parameters that enhance the accuracy of the three-dimensional (3D)-printed part. The 3D-printed dental casts, complete dentures, and single crowns were investigated for their accuracy. Similarly, digital implant surgical guides, implant fixed and removable prostheses, and removable partial dentures were all checked for accuracy to plan the best combination of the printing parameters [3,6,8,9,10,11,12,13,14,15].

Stereolithography (SLA) and digital light processing (DLP) techniques are examples of accurate vat-photopolymerization category besides selective laser melting (SLM) and selective laser sintering (SLS) from the powder bed fusion category [1]. Researchers studied the accuracy of these 3D printing techniques to clarify the influence of the 3D printing parameters [9,10,11,16,17,18,19]. In addition, the impact of various 3D printing factors on the printing outcome was also investigated [7,8]. The role of the build angle, layer thickness, support structure diameter, density, and tip size on the printed part were all considered [7,8,9,11,18,20,21]. Both DLP and SLA technologies are popular manufacturing techniques used in the prosthetic labs for producing RPD frameworks. Ease of application, availability, timesaving, and reasonable cost of both materials and printers encourage many labs to adopt these techniques over the conventional technique [6]. Although SLM technology could produce the final metallic RPD framework directly, this technology is not widely available in prosthetic laboratories and is mostly available in research labs. The accuracy of its manufactured RPD frameworks is also under study [18,19,22]. On the other hand, DLP and SLA are most common now in prosthetic labs and have largely replaced conventional RPD fabrication. Other techniques that fabricate final RPD from polymers such as nylon are still under observation [23].

Although accuracy is critical for 3D printing in the dental field, we should have a balance with other outcome settings, such as time of printing and material consumed [7,8]. Certainly, it is crucial to reduce printing time and the amount of material used, but not at the expense of the accuracy limit desired [7,8]. Unfortunately, the accurate 3D printed part may involve more time and waste more materials [2]. Simulated optimization research was carried out to determine the best parameter combination for 3D printing medical models with minimal time and materials used [7].

To study the accuracy of the 3D-printed models, they were 3D scanned and compared with the original 3D model. This process was explained as the trueness of the 3D printed model and requires the use of specific tools and software to be examined quantitatively and qualitatively [5,6,8,10]. Another technique was applied by checking the fitness of the protheses on the underlying cast by matching between their surfaces and digitally measuring gap distance between reference points [3,6,24]. Studies also measured the internal fit through micro-computed tomography (µ CT) between the tested surfaces, followed by correlation to the trueness values [21,22,25]. Other less accurate examinations were considered such as the visual observation, clinical assessment, and checking the internal fit by silicone registration material [26].

After databases search, no study investigated the influence of the 3D printing parameters of the DLP technology on the RPD accuracy of fitness, printing time, and amount of material consumed. Therefore, the study aim was to examine the effect of build angle and support structures’ diameter of the 3D printed RPD frameworks to clarify the optimal settings based on fitness, time, and material consumption, and correlations between these outcome parameters. We could hypothesize that changing 3D printing parameters such as build angle and support structure diameter can enhance printing outcomes. Additionally, there is a correlation between accuracy, printing time, and material consumed.

## 2. Materials and Methods

**Figure 1:**
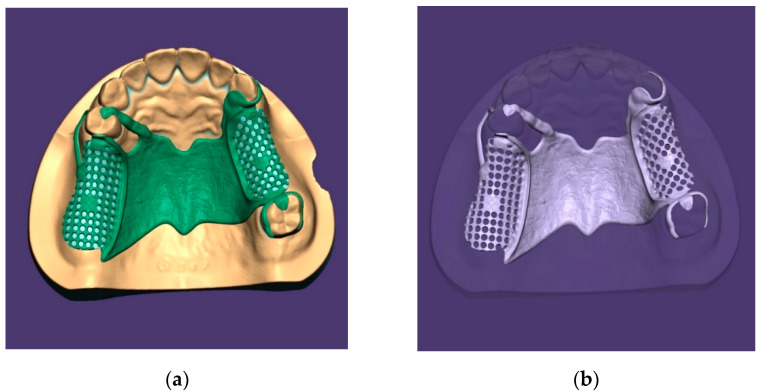
(**a**) Virtual waxing of the removable partial denture framework; (**b**) framework ready to export and three-dimensional printing.

A silicone replica (Dental Model; Nissin Dental, Kyoto, Japan) of partially edentulous maxillary arch, Kennedy class II modification 1, was poured by hard stone. The RPD framework design was drawn and the abutment preparation areas were marked. Following abutment preparation on the casts, three distinct shapes were drilled at the major connector area on the hard palate. The cast was 3D scanned using the desktop scanner (E4, 3Shape A/S; Copenhagen, Denmark) and was saved as a standard tessellation language (STL) file. The 3D model of the RPD framework was created using dental computer-aided design (CAD) software and 3D printing settings after processing multiple steps (Figure 1).

**Figure 2:**
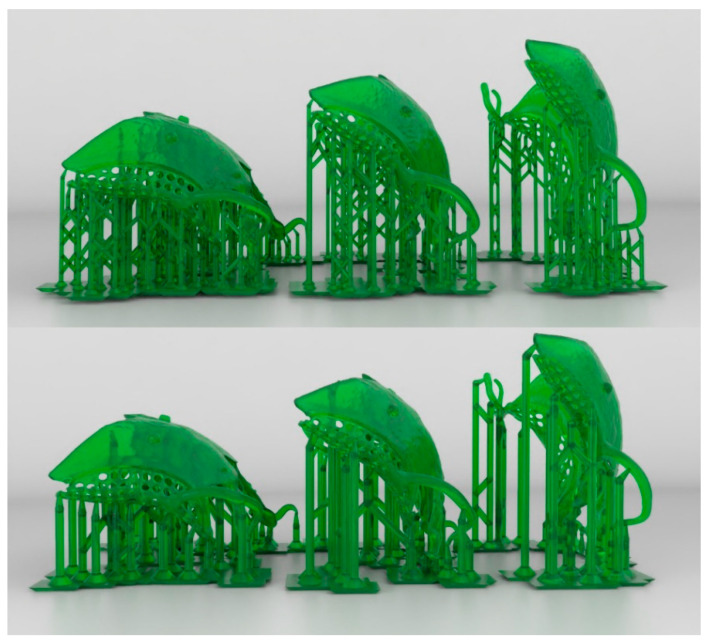
Image representative to the 3D printed parts at different build angles and support thicknesses. The upper three parts for the thin support with 110, 135, and 150-degree angles (from right to left). The lower parts showing same sequence at thick support.

After that, the RPD framework was 3D printed based on the studied parameters that were assigned to the 3D printing software (Chitubox Pro; CBD Ltd., Guangdong, China). Three different build angles (110°, 135°, and 150°) and two support structure diameters (fine = 1.2 mm and thick = 1.5 mm diameters) were studied to form six groups (Figure 2). To determine the number of samples, we performed a power analysis study by sample power analysis software (G*Power v3.1.9.4 software; Heinrich-Hein-University, Dusseldorf, Germany). The power sample data for means comparison was represented as (Total sample size = 60; effect size [*f^2^* (V)] = 0.5; actual power = 82.4%; power = 82%; α = 0.05). The sample size for the correlation study was denoted as (Total sample size = 21; Correlation ρ H1 = 0.64; Power (1-β err prob) = 0.90; Actual power = 91%; α = 0.05).

Based on the power analysis, 60 RPD frameworks were 3D printed (10 for each group). A castable resin material (DentaCast; ASIGA, Sydney, Australia) was used for fabricating the framework by the 3D printer machine (Max UV; ASIGA, Sydney, Australia). A post-printing treatment was pursued by 10 min of isopropyl alcohol (IPA) rinsing, drying, and then curing for 20 min in the post-curing chamber (Asiga Flash; ASIGA, Sydney, Australia). The previous step was mandatory to polymerize the frames and get sufficient strength without distortion.

All supports were removed, and the frameworks were sprayed by antiglare spray (3D Scan Spray; Helling GmbH, Tornesch, Germany) before the 3D scanning process. A pre-customized silicone key was prepared to have a fixed standardized framework position during scanning. Scanning was carried out, and all files were labeled according to their group.

All framework files that came from scanning were edited using a 3D metrology software (Geomagic Control X; v 2018, 3D Systems, Rock Hill, SC, USA). All of the unnecessary areas were removed and only tissue-side surfaces that contact the cast surface were kept (areas of interest). All edited files were simultaneously aligned to the cast 3D surface, aided by the previously created reference shapes. After alignment, each frame surface was 3D compared to the cast surface (reference model). The root-mean-square (RMS) values were recorded at a critical value (±300 µm) and tolerance (±50 µm). All values of the time and material consumed during the printing process were recorded as received from the 3D printer and labeled according to their testing group [8].

Statistical analysis was calculated by statistical analysis software (IBM SPSS Statistics, v21.0; IBM Corp., Armonk, NY, USA) to study the combined effect of building angle and build-structure diameter on the accuracy of fitness, printing time, and material consumption. The data were checked for normality and homogeneity of variance. The one-way analysis of variance (ANOVA) test was used to check the difference between groups in printing time and material consumption variables, followed by a post-hoc test (Bonferroni’s with adjustment). The accuracy of fitness data did not show homogeneity of variance (Levene’s value= 3.907, sig. = 0.004). Consequently, Welch’s test was applied, followed by a post-hoc test for inhomogeneous variables (Games-Howell test). Correlations between the three continuous variables were also calculated using Pearson’s correlation test. The alpha value was (α = 0.05) for all tests.

## 3. Results

The 150-degree and small diameter supports group (150-DS) expressed the highest accuracy among other groups by showing the least mean of the root-mean-square value (0.061 ± 0.015 mm). In contrast, the 110-degree and large diameter supports group (110-DL) was the least accurate groups by having the highest mean of the RMS (0.098 ± 0.014 mm) among all groups with statistically significant difference from groups (150-DS), (135-DS), (150-DL) and (135-DL) at (*p* < 0.001), (*p* = 0.002), (*p* = 0.03) and (*p =* 0.003), respectively (Table 1). The RMS of (135-DS) was less than (135-DL) followed by (150-DL), (110-DS) and (110-DL), respectively, with no significant values among these groups (*p* < 0.05), (Table 1).

The results of the printing time (PT) showed that the least mean value was for (150-DS) group (172 ± 2 min), with a statistically significant difference (*p* < 0.001) from groups (135-DS), (110-DS), (135-DL), (110-DL). Group (110-DL) required significantly more (PT) than all other groups (*p* < 0.001) except for group (110-DS). All groups of the same build angle and different support diameters showed statistically insignificant differences (*p* ˃ 0.05). The printing time of groups (150-DS) and (150-DL) was matched (172 ± 5 min). Similarly, the printing time of groups having the same angle (135-DS), (135-DL), and (110-DS), (110-DL) showed similar values (186 ± 2 and 201 ± 3 min), respectively (Table 1).

The mean value of the (110-DS) group showed the lowest material consumption (MC) (2.39 ± 0.30 mL), with statistically significant differences from all other groups (*p* < 0.001). In contrast, the group (150-DL) significantly consumed more materials than any other group (5.26 ± 0.14 mL) (*p* < 0.001) (Table 1).

There was a linear relationship observed between PT and RMS (Pearson’s correlation = 0.479, *p* < 0.001). A negative correlation was seen between PT and MC (Pearson’s correlation = −0.668, *p* < 0.001). There was no correlation observed between RMS and MC (Pearson’s correlation= −0.190, *p* = 0.146) (Table 2).

**Figure 3:**
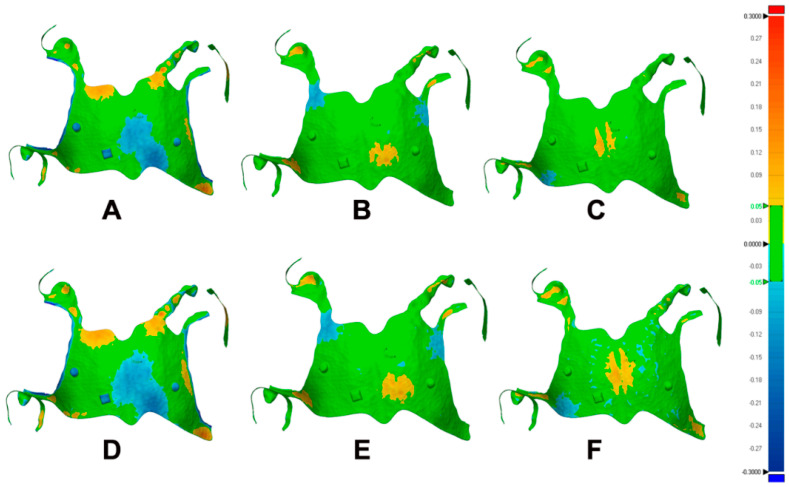
Sample of color maps of the three-dimensional deviations of different groups generated by the metrology software with a color-coded scale showing level of deviations. (**A**–**C**) represent 110, 135, and 150-degree angles with thin support structures. (**D**–**F**) represent same angle sequence but with thick support structures.

The sample of color maps of the frameworks’ 3D deviations could be seen in Figure 3. Based on the color scale considered, green color will represent the most fitted and accurate part within the tolerance selected. The darker the blueish color, the more negative deviation is the surface and the less fitness and accuracy. In contrast, the more red the surface is, the more positive the surface is deviated, which also expresses less fitness and accuracy (Figure 3).

The color maps of (110-DS) and (110-DL) groups displayed areas of negative deviations (bluish color) in the mid-posterior part of the major connector extended to the middle area. In addition, some islands of positive deviations (yellow to orange) were seen scattered at the periphery of the frameworks, especially at the anterior borders and the minor connectors (Figure 3A,D). The color maps of (135-DS) and (135-DL) groups showed more homogenous deviations with near central positive deviation (yellow color) at the major connector and the minor connectors. Few islands of negative deviation (light blue) were seen at the periphery (Figure 3B,E). For groups (150-DS) and (150-DL), small central positive deviation in the major connector and the minor connector, and traces of negative deviation were seen (Figure 3C,F). Group (150-DS) had the most homogenous color deviation (more green color) among all other groups. Overall, the large diameter support groups exhibited more intense color maps than the small diameter groups, regardless of the deviations’ locations.

## 4. Discussion

The results of the study reported that there was a significant decrease in the accuracy of frameworks manufactured at a 110-degree angle and large diameter supports. Moreover, significant differences in printing time were shown among groups with different build angles regardless of their support diameters. All groups displayed significant differences in the amount of material consumed. Therefore, the first hypothesis was partially accepted. The results also showed a positive correlation between RMS and the printing time and another negative correlation between printing time and material consumed. No correlation was reported between RMS and material consumed. Accordingly, the second hypothesis could also be partially accepted.

Although there was an improvement in the accuracy of the frameworks created at higher build angles and small diameter supports (Table 1) and (Figure 3A), only frameworks created at a 110-degree angle and large diameter support have significantly low accuracy (Table 1) and (Figure 3D). Building the RPD framework at a 150-degree angle and small diameter support showed the lowest RMS and so was the most accurate (Table 1) and (Figure 3C). This finding agreed with another study regarding support diameter, which showed better accuracy with thinner support diameter [10]. Our results also were in accordance with another study performed on 3D printed complete dentures, as they reported an insignificant change in accuracy between most of the build angles tested [9]. However, many studies recommended the use of a 135-degree build angle for fabrication of the 3D printed complete dentures, surgical guides, and crowns [8,9,13,17].

The results of the printing time revealed a significant change among different build angles with the least time required for 150-degree angle groups and longest time in 110-degree angle groups (Table 1). This finding agreed with Rubayo et al.’s [8] study. They confirmed an increase in the printing time as the build angle comes closer to an upright position. In the same context, our results coincide with another study, which showed an increase in printing time by changing the build angle closer to 90 degrees [7,10]. Another finding was that changing support diameter did not affect the printing time in the same build angle. There was a matching between printing time of frameworks at the same build angle and different support structure thickness while more resin consumed with the thick support structure (Table 1). This could be attributed to the rate of printing in the support section may differ from that of the framework part. Generally, more accuracy will be required for framework rather than the support structure. However, more resin was consumed to print the thick structure [7,8].

Material consumption was significantly reduced in build angle 110-degree with small diameter support structures and increased as the angle increased (Table 1). This finding matched the results of other studies [7,8]. However, they showed that type of model and other parameters such as support structure density, tip size, printing layer thickness and build orientations may influence the material consumption [7].

The results of the current study mostly attributed to the influence of the printing time on all other parameters. The closer the build orientation was from an upright position, the more printing time required and so the more time the framework was kept in the printing machine in an immature condition [2,8]. This makes the framework more vulnerable to distortion before full polymerization in the post-curing chamber. Consequently, more 3D deviation expressed as higher RMS value and seen as negative (blue color) deviations bands in the color maps extended from the major connector periphery to the central area. It should also be mentioned that thinner support showed more homogenous color maps, which could be clarified as a more suitable support for such types of objects (Figure 3). In the same context, group 110-degree consumed less material since less of the support structure was required, which may predispose the group to more distortion (Table 1).

Three-dimensional printing outcomes of the RPD reflect the mutual effect of root-mean-square, printing time and material consumption values. This was confirmed by the linear positive relationship between root-mean-square and printing time values (Pearson’s correlation = 0.479) and the linear inverse relationship between printing time and material consumption values (Pearson’s correlation = −0.668) (Table 2). This means that the further the build angle from 90-degree and the thinner support structures, the higher the accuracy and the lesser the printing time (albeit at the expense of material consumed) [8]. Although the optimal condition is achieved by good accuracy in a shorter time and with lesser material consumption, this condition is hard to accomplish. Usually, the accuracy in minimal printing time is more desirable. To summarize, we believed that printing time was the key element of the study’s findings.

The current study had some limitations that could be addressed. Some other printing settings that may influence the printing outcomes could be considered. These settings, such as support structure density, tip size and shape, as well as printing layer thickness, may interact with the printing outcomes [1,2,7]. Testing one 3D printing technology (DLP) and one material (castable resin) could be recognized as limitations. These limitations could initiate new insights and topics for future studies.

## 5. Conclusions

It could be concluded that the build angle and diameter of the support structures have an impact on the (DLP) three-dimensional printing outcomes. This impact could be beneficial to optimize the accuracy, printing time, and material consumption. Based on the results, building the removable partial denture framework at a 150-degree angle and thin diameter support structures showed the optimal setting as an accurate and fast printing setting to be used regardless of the material consumed. Although the printing time of the same build angle was similar, more resin was consumed for printing thick supports.

## Figures and Tables

**Table 1:** Mean and standard deviations of root-mean-square (RMS) in (mm), printing time (PT) in minutes (min) and material consumption (MC) in milliliter (mL) of studied groups.

Groups	RMS (mm)	PT (min)	MC (mL)
150-DS	0.061 ± 0.015 ^a^ *	172 ± 2 ^a^	3.88 ± 0.16 ^a^
135-DS	0.069 ± 0.014 ^a^	186 ± 2 ^b^	3.34 ± 0.27 ^b^
110-DS	0.088 ± 0.025 ^a,b^	201 ± 2 ^c^	2.39 ± 0.30 ^c^
150-DL	0.076 ± 0.015 ^a^	172 ± 5 ^a^	5.26 ± 0.14 ^d^
135-DL	0.074 ± 0.007 ^a^	186 ± 3 ^b^	4.82 ± 0.21 ^e^
110-DL	0.098 ± 0.014 ^b^	201 ± 3 ^c^	3.46 ± 0.29 ^b^

* Groups with different letters in the same column are statistically different, *p* ≤ 0.05.

**Table 2:** Results of Pearson’s correlation values and significance for correlations between root-mean-square (RMS), printing time (PT), and material consumption (MC).

		PT	MC
RMS	Pearson Correlation	0.479	−0.190
	Sig. (2-tailed)	<0.001 *	0.146
PT	Pearson Correlation	1	−0.668
	Sig. (2-tailed)		<0.001 *

* Significant at *p* < 0.05.

## Data Availability

The data presented in this study are available on reasonable request from the corresponding author.
